# Growth-inhibitory and cell cycle-arresting properties of the rice bran constituent tricin in human-derived breast cancer cells *in vitro* and in nude mice *in vivo*

**DOI:** 10.1038/sj.bjc.6602124

**Published:** 2004-08-17

**Authors:** H Cai, E A Hudson, P Mann, R D Verschoyle, P Greaves, M M Manson, W P Steward, A J Gescher

**Affiliations:** 1 Cancer Biomarkers and Prevention Group, Department of Cancer Studies and Molecular Medicine, University of Leicester, Leicester LE2 7LX, UK; 2 Medical Research Council Toxicology Unit, University of Leicester, Leicester LE1 9HN, UK

**Keywords:** chemoprevention, cell cycle arrest, flavonoids, nude mice, rice bran

## Abstract

Tricin, a flavone found in rice bran, inhibits the growth of human-derived malignant MDA-MB-468 breast tumour cells at submicromolar concentrations. As part of the exploration of tricin as a potential cancer chemopreventive agent, we investigated the duration and cell cycle specificity of growth inhibition elicited by tricin *in vitro* and the effect of tricin on the development of MDA-MB-468 tumours grown in immune-compromised MF-1 mice *in vivo*. Preincubation of MDA-MB-468 cells with tricin (1–40 *μ*M) for 72 h compromised cell growth after tricin removal, and such irreversibility was not observed in human breast-derived nonmalignant HBL-100 cells. Tricin (⩾5 *μ*M) arrested MDA-MB-468 cells in the G2/M phase of the cell cycle without inducing apoptosis as adjudged by annexin V staining. In nude mice consumption of tricin with the diet (0.2%, w w^−1^) from 1 week prior to MDA-MB-468 cell implantation failed to impede tumour development. Steady-state levels of tricin in plasma, breast tumour tissue and intestinal mucosa, as measured by HPLC, were 0.13 *μ*M and 0.11 and 63 nmol g^−1^, respectively. Cells were exposed to tricin (0.11, 1.1 or 11 *μ*M) *in vitro* for 72 h and then implanted into mice. The volume of tumours in animals bearing cells pre-exposed to 11 *μ*M tricin was less than a third of that in mice with control cells, while tumours from cells incubated with 0.1 or 1.1 *μ*M tricin were indistinguishable from controls. These results suggest that the potent breast tumour cell growth-inhibitory activity of tricin *in vitro* does not directly translate into activity in the nude mouse bearing the MDA MB-468 tumour. While the results do not support the notion that tricin is a promising candidate for breast cancer chemoprevention, its high levels in the gastrointestinal tract after dietary intake render exploration of its ability to prevent colorectal carcinogenesis propitious.

One of the perceived health benefits derived from consumption of cereal foods is their putative ability to prevent cancer (Jacobs *et al*, 1995; Kasum *et al*, 2002). Ingestion of whole grains and grain products has been shown in epidemiological studies to be associated with a reduced risk of cancers of the breast (Nicodemus *et al*, 2001), colon (Smigel, 1992) and prostate (Jain *et al*, 1999). Rice, predominantly the unpolished brown (bran-containing) variety, is a staple cereal food in Asia, where the incidence of cancer of the breast, colon and prostate is considerably lower than in Western countries. An extract of rice bran, which had undergone fermentation by *Aspergillus oryzae*, was shown to inhibit the formation of azoxymethane-induced aberrant crypt foci and tumours in rats (Katyana *et al*, 2002), consistent with a link between brown rice consumption and reduced cancer incidence. Recently, an attempt was made to characterise potential cancer chemopreventive properties of rice bran ingredients (Hudson *et al*, 2000). Among potentially pharmacologically active agents identified was the flavone tricin (4′,5,7-trihydroxy-3′,5′-dimethoxyflavone, for structure see Figure 1

**Figure 1:**
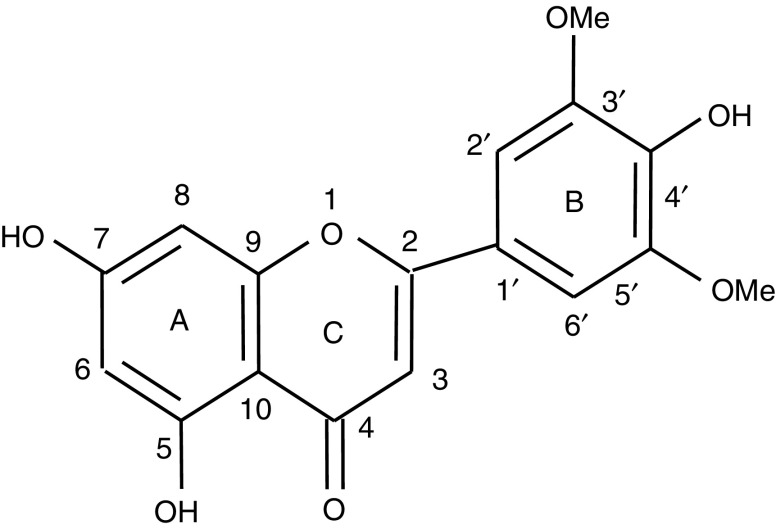
Chemical structure of tricin, 4′,5,7-trihydroxy-3′,5′-dimethoxyflavone.

). Tricin occurs as a glucoside in rice bran and other grass species such as wheat, barley and maize. It interfered potently with the growth of human-derived malignant MDA-MB-468 breast cancer cells, and was much less growth-inhibitory in HBL-100 cells, nonmalignant but transformed human breast cells (Hudson *et al*, 2000). In 1981 tricin was characterised in the plant *Wikstroemia indica* as a constituent with antileukemic properties in rodents (Lee *et al*, 1981). It needs to be pointed out though that the potential contribution of dietary tricin to the low incidence of cancer of the breast, colon or prostate in Asia as compared to the West is difficult to reconcile with its occurrence in cereal grains such as wheat, corn and barley, which are consumed in large amounts in the West. There is virtually no information available on the mechanisms via which tricin may exert cancer cell growth arrest. Flavonoids such as tricin are common in the plant kingdom, and several of them are considered to possess cancer chemopreventive efficacy. Thus, their pharmacological mechanisms are subject to much current research. The anticarcinogenic activity of flavonoids has been linked to antioxidation, because they are polyphenols with a high propensity to undergo redox reactions and to scavenge oxygen radical species (Rice-Evans *et al*, 1996; Mitchell *et al*, 1998; Havsteen, 2002). Other mechanisms that have been discussed include inhibition of the metabolic activation of carcinogens (Le Marchand, 2002), induction of enzymes that detoxify carcinogens (Talalay *et al*, 1995; Le Marchand, 2002) and anti-inflammation mediated via inhibition of cyclooxygenase or lipoxygenase enzymes or inhibition of inducible nitric oxide synthase (Raso *et al*, 2001). Furthermore, cellular signalling pathways, which regulate the cell cycle machinery and thus influence cell survival/proliferation and promotion and progression of malignant cells, are pharmacological targets of many flavonoids (Manson, 2003), exemplified by quercetin contained in onions and wine, genistein in soya and apigenin in leafy vegetables and fruits.

In the light of the exquisite sensitivity of human-derived MDA-MB-468 breast cancer cells towards the growth-inhibitory properties of tricin (Hudson *et al*, 2000), we wished to characterise the effect of tricin on breast cancer cells further, with the ultimate aim of exploring its chemopreventive potential. The following three hypotheses were tested: (i) tricin-induced cell growth inhibition is irreversible, that is, on incubation of cells in culture with tricin and its subsequent removal, cellular growth potential continues to be compromised; (ii) the growth-inhibitory activity of tricin is associated with a selective effect on the cell cycle, engendering increased apoptosis, and (iii) dietary administration of tricin to immune-compromised mice bearing the MDA-MB-468 tumour, which is sensitive to tricin *in vitro*, achieves target tissue levels sufficient to compromise tumour development *in vivo.* As we found that tricin in the diet (0.2%) had only a slight effect on tumour formation *in vivo*, an additional question was addressed as to whether growth arrest observed in cells exposed to tricin *in vitro* translates into reduced tumour development *in vivo* using an *in vitro*–*in vivo* bioassay. This experimental paradigm entails exposure of cells to tricin *in vitro* prior to subcutaneous implantation into mice, and assessment of the ability of the cells to develop into full-blown tumours *in vivo*. Overall, the work was designed to contribute to the database, which will eventually help to decide whether tricin is worthy of clinical development as a breast cancer chemopreventive agent.

## METHODS

### Chemicals

Tricin, kindly provided by Dr Izet Kapetanovic (Chemoprevention Agent Development Research Group, NCI Division of Cancer Prevention, Bethesda, MD, USA), was dissolved in DMSO to furnish solutions of 25 mM (for use in cell culture) and 0.1 mg ml^−1^ (for use in chemical analysis). Quercetin, which was used as internal standard in the HPLC analysis, RPMI-1640, DMEM, foetal bovine serum, Glutamax, and RNase A were purchased from Sigma-Aldrich Comp. Ltd. (Poole, UK). Fluorescein-isothiocyanate-conjugated annexin V was bought from Bender MedSystem (Vienna, Austria). Ammonium acetate, acetic acid, disodium ethylenediamine-tetraacetic acid (EDTA), and sodium dodecyl sulphate (SDS) were all of analytical grade (Sigma). HPLC-grade methanol was obtained from Fisher Chemicals Comp. (Loughborough, UK), HPLC-grade water was generated in-house in a Nano-Pure water purification system (Barnstead, UK).

### Cell culture and treatments

Human-derived MDA-MB-468 breast cancer cells, originally obtained from the American Type Culture Collection (Rockville, MD, USA), were cultured routinely in RPMI 1640, supplemented with 10% FCS and 2 mM Glutamax; human-derived HBL-100 breast cells, kindly provided by Professor R Walker (Department of Cancer Studies, University of Leicester) were cultured in DMEM, supplemented with 10% FCS. Details of growth conditions for both cell types have been described by Hudson *et al* (2000). Cells were cultured in a humidified atmosphere (95% air 5% CO_2_, 37°C). To aid with the interpretation of the cell growth data described below, it is important to note that the doubling times of the two cells were similar, 29 and 32 h for MDA-MB 468 and HBL 100 cells, respectively. Tricin stock solution was diluted with medium to the desired concentrations, so that the final DMSO content in culture medium was never greater than 0.2% (v v^−1^), which on its own did not affect cell-growth and cell-cycle distribution. Cells (seeded at 1 × 10^4^ per well, 12-well plates) were kept for 24 h to allow attachment to the substratum, after which tricin was added. After periods of up to 168 h cells were harvested (trypsinisation) and counted using a ZM model Coulter Counter (Beckman Coulter, Luton, UK). IC_50_ values were computed using plots of cell number (expressed as a percentage of control) *vs* tricin concentration.

To determine the ability of cells to recover following exposure to tricin, cells were treated for 72 h with tricin (1–40 *μ*M), after which they were maintained in fresh medium omitting tricin for a further 96 h. The ability of cells to recover following treatment was calculated as fold increase in cell number over that observed at 72 h. For comparison cultures were included in which treatment with tricin was continued for the whole 168 h period.

### Flow cytometric cell cycle analysis and assessment of apoptosis

Asynchronously growing cells were exposed to tricin (1–40 *μ*M) or vehicle control only for 24, 48, 72 or 96 h and harvested. For cell cycle analysis, cells were centrifuged, washed (phosphate-buffered saline, PBS) and fixed in ethanol (70%, 4°C, 12 h). Cells were centrifuged (600 × **g**, 10 min), and the pellet was resuspended and kept (4°C, 12 h) in PBS (1 ml) containing RNase (0.1 mg) and propidium iodide (PI, 5 *μ*g). Flow cytometric analysis was performed with a Becton Dickinson FACScan (Becton Dickinson UK Ltd, Cowley, UK). Cell cycle compartments were integrated using the ModFit LT software (Becton Dickinson). To distinguish apoptosis from necrosis, cells were treated as above, harvested, washed in PBS and resuspended in medium and incubated for 30 min (37°C). Cells were spun down and suspended in annexin V-staining buffer (1 ml). Cell suspension to which FITC-annexin V had been added was kept for 8 min (room temperature), after which PI was added and the mixture was incubated (2 min). Samples were analysed by flow cytometry using the CellQuest software (Becton Dickinson). For each sample the number of total events acquired and gated for analysis was 5000.

### Inoculation of MDA-MB-468 cells into mice and evaluation of the effect of tricin

Female MF1 athymic nude mice (purchased from Harlan, UK) were maintained under sterile and standard environmental conditions and used when they were 6–8 weeks of age. Animal experiments were carried out under a project license granted to Leicester University by the UK Home Office. The experimental design was vetted by the Leicester University Local Ethical Committee for Animal Experimentation and meets the standards required by the UKCCCR guidelines (Workman *et al*, 1998). Tricin was thoroughly mixed with RM1 powdered diet, and the mixture was kept at 4°C for up to 1 month. Diet in food hoppers was replaced once weekly. Groups of 12 mice each received either control diet (RM1) or RM1 diet supplemented with 0.2% tricin (w w^−1^) commencing 1 week before tumour inoculation. MDA-MB-468 cells (10^7^) suspended in Leibovitz medium (0.1 ml) were injected subcutaneously into the right flank. For the *in vitro*–*in vivo* bioassay, cells were exposed to tricin 0.11, 1.1 or 11 *μ*M (or the vehicle only) for 72 h, after which remaining adherent cells were harvested. Yields of adherent cells at the three different tricin concentrations were 100, 81 and 62%, respectively, of vehicle controls. An aliquot of the cell suspension (5 × 10^6^ cells) was then injected into mice. Body weights were checked weekly. Tumours were palpable 1 week after cell inoculation. Tumour size was assessed by calliper, and tumour volume was calculated by the formula:


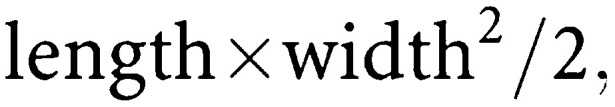


where length is the larger and width the smaller diameter of the tumour (in mm).

Tumour tissue was fixed in neutral buffered formalin and embedded in paraffin wax. Conventional histological sections, 5 *μ*m thick, were cut and stained with haematoxylin and eosin.

### HPLC analysis of tricin in cellular incubates and mouse plasma and tissues

Cells (0.5 × 10^6^ per 10 cm plate) were incubated with tricin, an aliquot of the medium (2 ml) was collected, and cells were harvested. The cell pellet was washed (PBS) and resuspended in water. At the end of the experiment mice were killed (cardiac exsanguination) under terminal anaesthesia with halothane. Blood was collected into heparinised tubes, and plasma was separated by centrifugation (2000 × **g**, 20 min, 4°C). Tissues (liver, tumour, small intestinal mucosa) were collected, the latter after extensive washing (PBS) to remove intestinal content, and stored at −80°C until analysis. Plasma and tissue samples were thawed at room temperature. Aliquots of cellular supernatant or plasma (0.1 ml) were vortex-mixed with internal standard, and analytes were extracted into an equal volume of acetone/acetic acid (0.1 M). The mixture was vortexed twice (1 min each) and centrifuged (300 × **g**, 20 min). An aliquot of the supernatant (0.2 ml) was mixed with water (0.1 ml). After centrifugation (5 min), a sample (50 or 100 *μ*l) was injected onto the HPLC column. Thawed tissue was washed with water to remove surface blood; excess water was blotted with filter paper. An aliquot (100–200 mg) of wet tissue was weighed, suspended in water (0.4 ml) and homogenised (manual glass homogeniser from Jencons, Leighton Buzzard, UK). Samples of plasma and livers from each mouse were analysed in duplicate, tumour tissue was pooled, and analysis of analyte in tumour was conducted in triplicate. The homogenate was extracted with an equal volume of acetone/acetic acid (0.1 M), as described for cellular incubates and plasma. Chemical analysis was conducted using an HPLC system consisting of a ProStar pump (Model 230) coupled with a ProStar Model 410 auto-sampler and a ProStar Model 310 UV-Vis detector, all from Varian (Varian Analytical Instruments, California, USA). Separation was achieved on a ThermoHypersil BDS C_18_ column (250 × 4.6 mm^2^, particle size 5 *μ*m, ThermoHypersil-Keystone, Cheshire, UK) using ammonium acetate buffer (0.1 M, pH 5.1) with methanol (52%) and EDTA (0.27 mM) as the isocratic mobile phase. The flow-rate was 1 ml min^−1^, the detection wavelength 355 nm. Development and validation of the HPLC analysis of tricin in the biomatrix have been reported before (Cai *et al*, 2003).

### Statistical evaluation

Data shown in the results section and the figures are expressed as means±s.d. Routine statistical evaluation with Minitab (v 13) software used either the general linear model ANOVA, with time and treatment as categorical variables followed by Fisher's least-square difference test, or one-way ANOVA following which statistical significance (*P*<0.05) was established by *post hoc* Tukey's pairwise comparison.

## RESULTS

### Reversibility of cell growth inhibition

**Figure 2:**
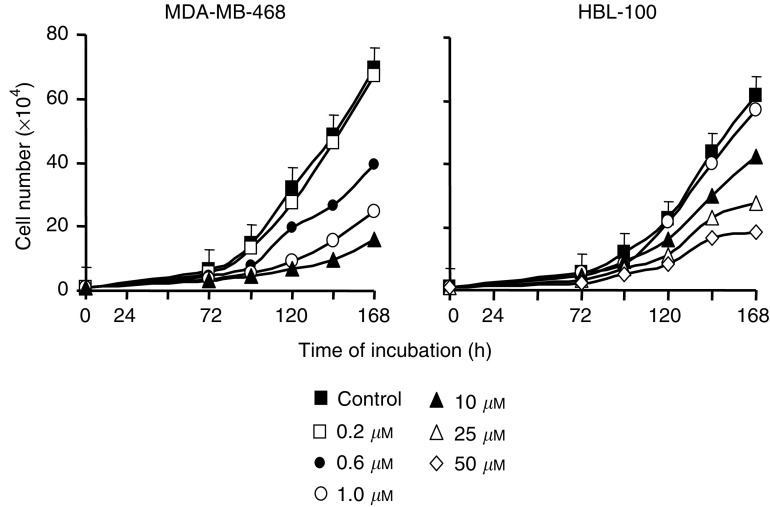
Effect of tricin on the growth of MDA-MB-468 and HBL-100 cells. Concentrations of 0.2–10 *μ*M were used with MDA-MB-468 cells, and 1–50 *μ*M with HBL100 cells. Cell numbers are the mean of triplicate samples. Pooled standard deviations are shown only in the case of numbers of untreated cells. Results are the mean of *n*=6, the values obtained at 120 h or later for tricin concentration of 0.6 *μ*M and higher in the MDA-MB-468 cells or of 10 *μ*M and higher in the HBL-100 cells were significantly different from the numbers of control cells (*P*<0.05) as determined by one-way ANOVA followed by Tukey's *post hoc* test. For details of cell culture and tricin treatment see Materials and Methods.

Initially, the breast cell growth-inhibitory properties of tricin were re-investigated in malignant MDA-MB-468 and nonmalignant, but transformed, HBL-100 cells. Cells were treated with tricin (0.1–50 *μ*M) for periods of up to 168 h, after which cells were counted. Tricin inhibited growth in a concentration- and time-dependent manner (Figure 2

). The IC_50_ values determined at 168 h for MDA-MB-468 and HBL-100 cells were 0.7 and 21 *μ*M, respectively, suggesting that tricin was 30 times more potent as a growth inhibitor in MDA-MB-468 than in HBL-100 cells, which is consistent with results reported earlier (Hudson *et al*, 2000).

**Figure 3:**
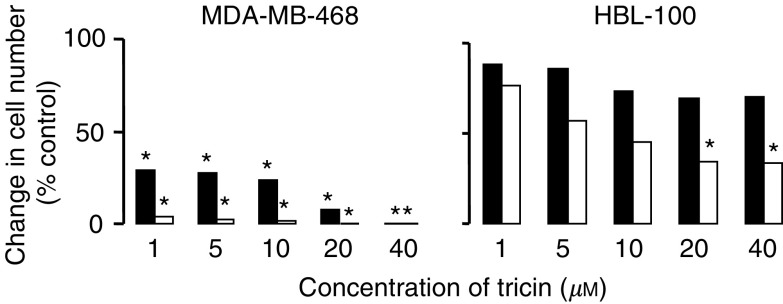
Recovery of MDA-MB-468 and HBL-100 cells following treatment with tricin. Cells were treated with tricin for 168 h (open bars), or treated with tricin for 72 h and then kept for 96 h in medium omitting tricin (‘recovery’, closed bars). Cells cultured without tricin for the whole of the 168 h incubation period were included as controls. Values reflect the increase in cell number under the respective conditions at the end of the experiment over cell numbers observed at 72 h, and they are expressed as percentage of the fold increase in control cells from which tricin was omitted for the whole 168 h. Results are the mean±s.d. of *n*=3–5, the pooled s.d. was 12.7 and 12.5% of the mean for MDA-MB-468 and HBL-100 cells, respectively. Star indicates that cell numbers were significantly different from those in control cultures (*P*<0.05) as determined by the ANOVA general linear model followed by Fisher's least significant difference evaluation. For further details of cell culture and tricin treatment see Materials and Methods.

In order to determine whether growth inhibition elicited by tricin was transient or permanent, the ability of cells to recover was determined following treatment of cells with tricin for 72 h, followed by exposure to tricin-free medium for another 96 h (‘recovery conditions’), while control cells were incubated without tricin for the whole 168 h period. For comparison, cultures were included in which cells were exposed to tricin for 168 h. After exposure to tricin 1–10 *μ*M for 72 h, followed by the recovery period, numbers of MDA-MB 468 cells were 25–28% of untreated controls. Only 8% overcame the inhibitory effect of exposure to 20 *μ*M tricin. Cells, which had been exposed to 40 *μ*M tricin, did not recuperate at all from tricin-induced growth arrest (Figure 3

). In contrast, when tricin was removed from the medium of HBL-100 cells, most cells recovered and grew at a rate close to that of control cultures.

### Effect of tricin on cell cycle distribution

**Figure 4:**
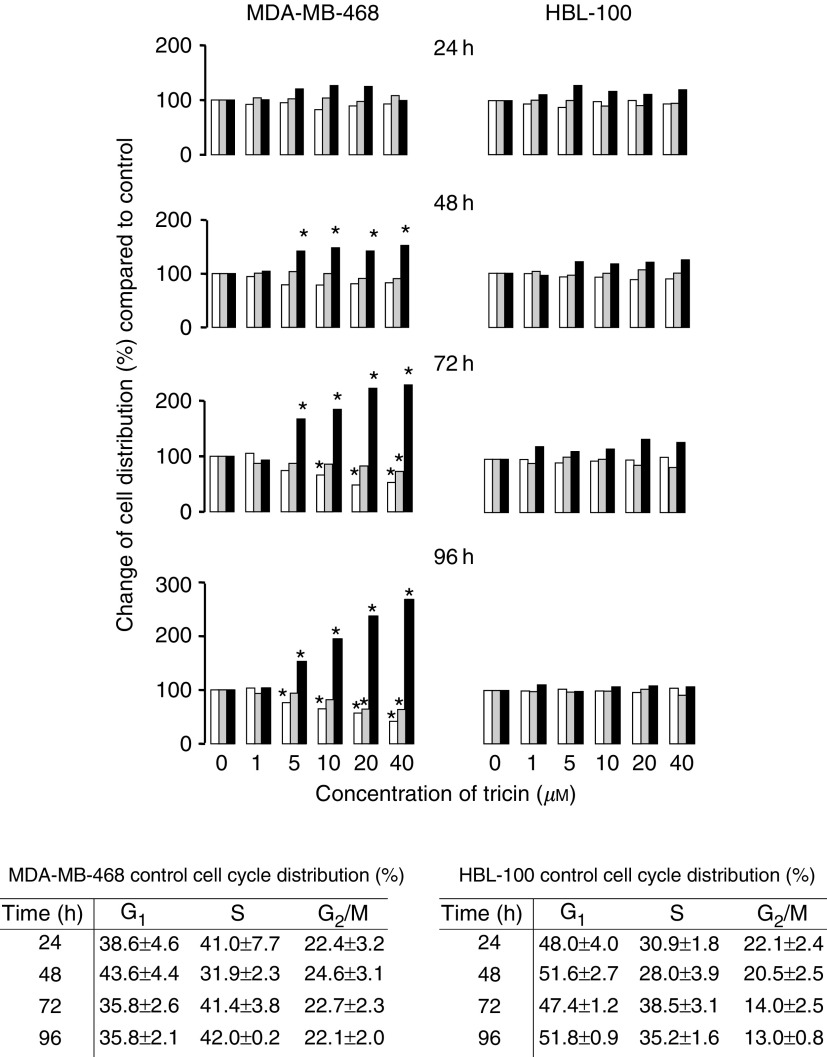
Effect of tricin on distribution of MDA-MB-468 and HBL-100 cells in the G1 (open bars), S (grey bars) and G2/M (closed bars) phases of the cell cycle. Cells were analysed by flow cytometry following treatment with tricin or vehicle control for 24, 48, 72 or 96 h and staining with PI. Values (mean of *n*=4) reflect the change in percentage cell number under the respective conditions from the number in that phase in control cultures at each time point. The s.d. was between 0.64 and 8.7% of the mean. The tabular inserts show cell cycle distribution observed in control cultures expressed as percentage of total cells (100%), and the values are the mean±s.d. of *n*=4. Star indicates that cell cycle distribution was significantly different from that observed for control cultures (*P*<0.05, determined by the ANOVA general linear model followed by Fisher's least significant difference). For details of cell culture, tricin treatment and flow cytometry see Materials and Methods.

The hypothesis was tested that the growth-inhibitory effect of tricin is the result of a perturbation of the cell cycle, possibly leading to programmed cell death. The effect of tricin on MDA-MB-468 and HBL-100 cell cycle distribution and level of apoptosis was explored. Tricin induced a G2/M phase arrest in asynchronous MDA-MB-468 cells, evident at concentrations ⩾5 *μ*M (Figure 4

). The tricin-mediated increase in percentage of cells in G2/M was dose- and time-dependent and largely attributable to depletion of cells in G1, with a decrease in S phase cells at the 72 and 96 h time points. In contrast, treatment of HBL-100 cells with tricin using the same concentration range and treatment times did not alter cell cycle distribution significantly (Figure 4).

Flow cytometric characterisation of phosphatidyl serine externalisation using FITC-conjugated annexin V and PI staining did not reveal any difference in detectable numbers of apoptotic cells between tricin-exposed and unexposed cells in either cell line at times from 24 up to 96 h. At 168 h there was a slight increase (10%) in combined numbers of apoptotic and necrotic cells in MDA-MB-468 cells treated with 40 *μ*M compared with control cells, but not in the HBL-100 cell line (data not shown).

### Stability of tricin in cell culture

Tricin is an agent for which hardly any pharmaceutical information exists. To permit interpretation of the effects on cell growth described above, we established whether it is stable under cell culture conditions. Tricin was analysed by HPLC in extracts of cells and culture medium after treatment for 72 h. Tricin was detectable in cultures when added at 5 *μ*M or more. Consistently, between 94% (cultures with 5 *μ*M) and 71% (at 40 *μ*M) of tricin added to the medium was recovered from cultures, irrespective of whether they contained MDA-MB-468 or HBL-100 cells, suggesting that stability during the period of cell culture incubation is satisfactory. There was no product of tricin degradation detected by the HPLC analysis.

### Effect of tricin on MDA-MB-468 tumour growth in nude mice *in vivo*

**Figure 5:**
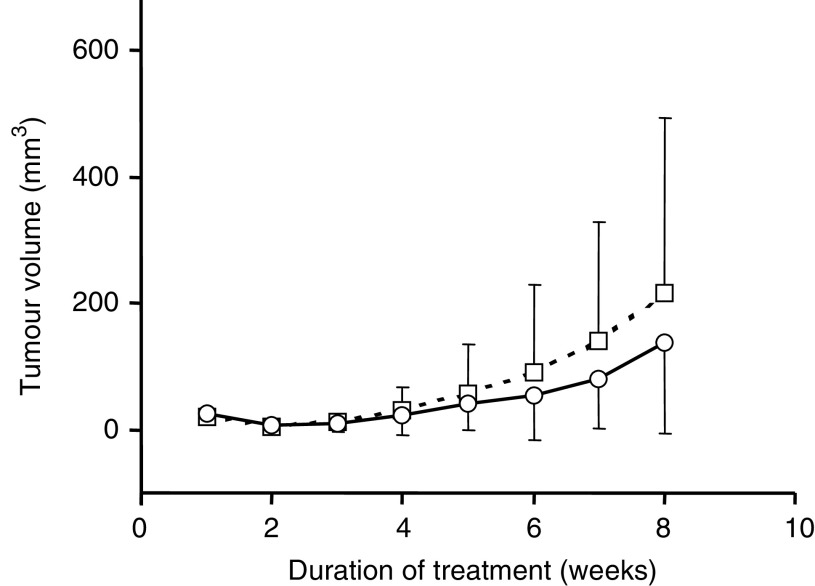
Effect of dietary supplementation with tricin (0.2%, circles) or control diet only (RM1, squares) on the growth of MDA-MB-468 tumour cells implanted in female MF1 nude mice. Tricin was added to the diet from 1 week prior to tumour implantation, continuing until termination of the experiment. Cells (10^7^) were implanted subcutaneously into the right hind flank. The results are the mean±s.d. for 12 mice. For details of cell culture, tumour inoculation and treatment see Materials and Methods.

In order to investigate whether the growth-inhibitory efficacy of tricin established in cells *in vitro* could also be observed *in vivo*, athymic nude mice harbouring MDA-MB-468 tumour cells as a subcutaneous injection received tricin at 0.2% in the diet (approximately 300 mg kg^−1^ body weight per day) from 1 week prior to tumour implantation to the end of the experiment. The body weight of mice on the tricin-supplemented diet did not differ from that of control mice, which suggests that tricin at this dose was easily tolerated. The tumours, which grew on the flanks of the mice, had features of cellular adenocarcinomas, with poor glandular differentiation. Tumours infiltrated connective tissue, skeletal muscle or lymphoid tissue. Figure 5

shows that in treated animals tumours grew at a rate that was slightly impeded compared to controls; nevertheless, the difference was not significant. Terminal tumour weights were 0.12±0.17 g in untreated and 0.11±0.15 g in treated animals (*n*=12 for each group). There was no clear histopathological difference in tumour cytology between the treated and control groups.

### Levels of tricin in mice *in vivo*

The distribution of tricin in the plasma and selected tissues was determined by HPLC at the end of the experiment, that is, after 9 weeks of dietary consumption of tricin (0.2%). Steady-state concentrations of tricin in the plasma, liver, small intestinalucosa and tumour tissue were 0.13±0.16 *μ*M and 0.80±0.36, 62.6±30.5 and 0.11±0.13 nmol g^−1^, respectively (*n*=12 for plasma, small intestine mucosa and liver and *n*=3 for tumour). These results suggest that tumour levels of tricin achieved by dietary consumption were insufficient to elicit tumour growth inhibition, as they were only a seventh of the IC_50_ established when MDA-MB-468 cells were grown *in vitro* (vide supra).

### Effect of preincubation of MDA-MB-468 cells with tricin on tumour growth *in vivo*

**Figure 6:**
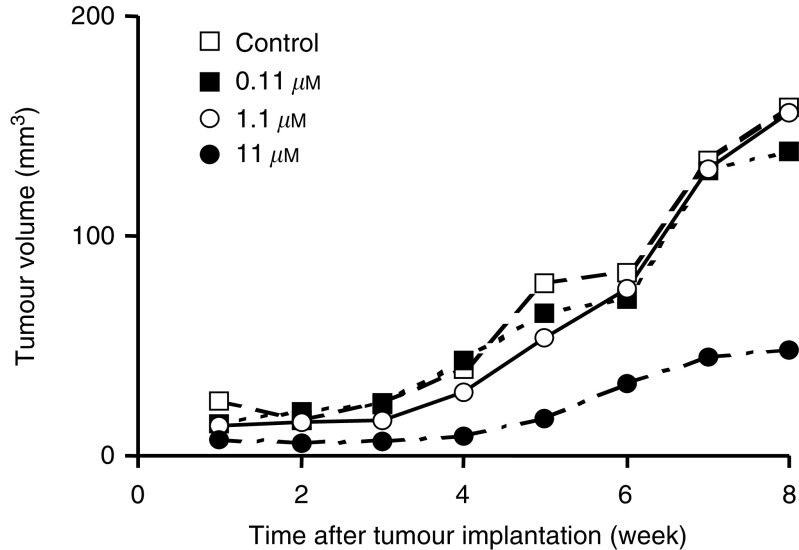
Effect of exposure *in vitro* of MDA-MB-468 tumour cells to tricin (0.11, 1.1 or 11 *μ*M) or to vehicle only on cell growth *in vivo* after implantation of cells into female nude MF-1 mice. In this *in vitro*–*in vivo* bioassay experiment, cells were incubated with tricin for 3 days, after which they were washed and implanted into the right hind flank of mice. Values are the mean for six mice. The s.d. values, which have been omitted for the sake of clarity, were consistently between 90 and 130% of the mean. For details of cell culture, cell incubation and tumour inoculation see Materials and Methods.

In the light of the lack of efficacy of tricin when administered with the diet at 0.2%, the tricin concentration required to compromise the development of the MDA-MB-468 tumour in an *in vitro–in vivo* bioassay setting was investigated. Under these conditions cells are exposed to tricin *in vitro* and subsequently transplanted into the animal. MDA-MB-468 cells were incubated with tricin, or with vehicle only, for 3 days, and then implanted into the flank of mice. The tricin concentrations employed in this experiment were 0.11, 1.1 and 11 *μ*M, that is, levels that were identical with, or ten and a hundred times greater than those concentrations to which MDA-MB-468 tumour had been exposed under *in vivo* condition when animals received tricin with the diet. Cells were still capable of proliferation when incubated with such concentrations of tricin *in vitro* as shown in Figure 2 (compare cell numbers at 72 and 168 h), and also showed some recovery of growth rate when tricin was removed from the culture medium following a 72 h incubation (Figure 3). Figure 6

shows that tumour volumes in mice, that had been inoculated with cells after incubation with 0.11 or 1.1 *μ*M tricin, were similar to those of tumours in mice, which had received cells incubated with the vehicle only. Nevertheless, mean tumour volume in animals bearing cells, which had been incubated with 11 *μ*M tricin, was less than a third of that in mice which had received control cells (Figure 6). The variation in tumour size in each of the four experimental groups of mice was substantial (see legend to Figure 6), an observation which is consistent with previous experience in this laboratory with MDA-MB-468 cells grown in the nude mouse model when used in the evaluation of other putative chemopreventive agents. The large variability confounded the establishment of statistical significance when tumour volumes were compared using either one-way ANOVA, Student's *t*-test or Mann–Whitney nonparametric test as statistical vehicle, irrespective of whether mice that failed to develop tumours were included or excluded. As some of the tumour size data were not normally distributed because of skewing consequent to very large or totally absent tumours, descriptive statistical evaluation of the data was performed yielding median values and interquartile ranges (Table 1

**Table 1:** Median values and interquartile ranges of tumour volumes in mice harboring MDA-MB-468 tumour cells previously exposed to tricin *in vitro*

**Week of tumour development**	**Cell pretreatment with tricin (*μ*M)**	**Median value (mm^3^)**	**Interquartile range (mm^3^)**
4	0	15	93
	11	2	17
6	0	45	173
	11	9	60
8	0	78	358
	11	8	88

a Cells were incubated for 72 h prior to inoculation into mice. For details of bioassay procedure, see Materials and Methods.

). Consistent with the results obtained when tumour volume was measured, terminal tumour weights in mice which had been inoculated with unexposed cells or with cells previously exposed to tricin at 0.11, 1.1 or 11 *μ*M were 0.083±0.052, 0.095±0.061, 0.071±0.084 and 0.037±0.084 g, respectively. Analogous with the dietary intervention study, there was no histopathological difference in cytology between tumours originating from mice, which harboured vehicle-exposed or tricin-exposed cells.

## DISCUSSION

The results outlined above allow the following four new insights into the pharmacology of tricin in breast cancer cells: (i) tricin arrests the growth of malignant MDA-MB-468 cells in an irreversible manner without exerting such an effect on nonmalignant HBL-100 cells; (ii) tricin arrests MDA-MB-468 cells in the G2/M phase of the cell cycle, but this inhibition is not the prelude to induction of apoptosis; (iii) the growth-inhibitory potential of tricin translates into antitumour efficacy in an *in vitro*–*in vivo* bioassay system, in which mice are inoculated with MDA-MB-468 cells that have been previously exposed to tricin; (iv) consumption of tricin with the diet at 0.2% is insufficient to elicit an antitumour effect in mice bearing the MDA-MB-468 tumour.

The involvement of G2/M phase arrest in the inhibition of breast cancer cell growth by tricin implicates a number of protein regulators of cell-cycle transition at G2/M checkpoints as potential candidate targets with which tricin might engage in causing growth arrest. Among these molecules are p34^cdc2^, Wee1, cdc25 and p21^cip^. p34^cdc2^ is a cyclin-dependent kinase, which in association with cyclin B_1_ controls transition of the cell from G2 into M phase. The kinases Wee1 and cdc25 inhibit and activate, respectively, the p34^cdc2^–cyclin B_1_ complex. p21^cip^ is a cyclin-dependent kinase inhibitor, which associates with the p34^cdc2^–cyclin B_1_ complex and promotes a block in both G2 and G1 phases. Apigenin (4′,5,7-trihydroxyflavone) is chemically closely related to tricin, its chemical structure differing from that of tricin only in that it lacks the two *O*-methyl moieties in positions 3′ and 5′ of ring B in tricin. Apigenin has also been shown to arrest MDA-MB-468 cells in G2/M phase, and this effect was linked to decreased expression of cell cycle proteins CDK1, CDK4, cyclins B_1_, D_1_ and A (Yin *et al*, 2001). Future studies will help to discover whether tricin interferes with cell-cycle regulatory proteins in a manner analogous to apigenin. The robust ability of tricin to confound breast cell proliferation hints at its potential as a chemotherapeutic agent, which might be a topic worthy of further elucidation, perhaps in judicious combination with a cellular signalling inhibitor.

The IC_50_ for MDA-MB-468 cell growth inhibition by tricin under conditions of cell culture for 168 h was 0.7 *μ*M. Similarly, incubation with 1 *μ*M tricin for 72 h sufficed to cause partially irreversible growth arrest when cells were allowed to grow *in vitro* subsequent to tricin removal. In contrast, in the *in vitro*–*in vivo* bioassay system, incubation of cells with 10 *μ*M tricin for 72 h prior to implantation into mice was required to afford a robust decrease in subsequent tumour development *in vivo*, whereas incubation with 1 *μ*M tricin failed to cause a marked decrease in tumour volume when compared to tumours in control mice. It seems that the murine subcutaneous flank environment, unlike the *in vitro* cell culture medium, provides factors that can overcome the partial disturbance by 1 *μ*M tricin of the mechanistic cascade regulating tumour cell proliferation. This difference in tricin concentration required for tumour growth arrest illustrates a discrepancy in intrinsic growth potential between cells in culture on the one hand and cells in the rodent organism *in vivo* on the other, even without taking differential tricin exposure characteristics and bioavailability between the two environments into account. For the sake of comparison, in an *in vitro*–*in vivo* bioassay similar to the one employed here with tricin, exposure of MDA-MB-468 cells to the isoflavone genistein at 30 *μ*M for 6 days prior to inoculation of cells into nude mice, abrogated tumour development completely, whereas incubation for 2 days affected tumour growth *in vivo* only ephemerally (Constantinou *et al*, 1998).

The result of the analysis of tricin in blood and tissues of mice, which had received the tricin-containing diet, intimates that the systemic bioavailability of tricin might be low when administered in this manner. Dietary administration led to comparable concentrations in plasma and tumour, in the 10^−10^–10^−9^ mole per ml or g range, which is clearly subefficacious when compared to concentrations required for growth inhibition *in vitro*. Thus, it is not surprising that supplementation of the diet with tricin failed to affect tumour development. Nevertheless, steady-state levels of tricin in the liver and gastrointestinal mucosa were six- and 450-fold, respectively, higher than the levels determined in the plasma, which suggests that it would be appropriate to explore the activity of dietary tricin in the prevention of hepatic and – in particular – gastrointestinal malignancies in rodents. Consistent with this notion, human derived SW480 colon cancer cells were found to be sensitive to the growth-arresting properties of tricin with an IC_50_ of 16 *μ*M (Hudson *et al*, 2000). As a tri-phenol tricin is conceivably a good substrate of conjugating enzymes, and efficient biotransformation may curtail its boavailibility. Preliminary evidence suggests that tricin does indeed undergo metabolic sulphation in the gastrointestinal tract and glucuronidation in mouse liver homogenate fortified with UDP glucuronyl transferase, yielding in each case two products identifiable by HPLC (Cai, Steward and Gescher, unpublished).

Good efficacy, lack of toxicity and reasonable bioavailability are three crucial prerequisites for the advancement of flavonoids into clinical development as cancer chemopreventive agents. In terms of the stage of clinical development of naturally occurring flavonoid aglycones, genistein is probably the molecule furthest advanced, as it is currently under phase II clinical evaluation for prevention of recurrent localised prostate cancer subsequent to radical prostatectomy (see website http://www.cancer.gov/clinical trials). Genistein has shown chemopreventive activity in several rodent models of carcinogenesis including those of the breast (Lamartiniere *et al*, 1995a; 1995b; Constantinou *et al*, 1996), but it is characterised by a very short plasma half-life in rodents (Supko and Malspeis, 1995). As far as the toxicity of genistein is concerned, its ability to induce site-specific DNA cleavage in the breakpoint cluster region of the *mixed lineage leukaemia* (*MLL*) gene *in vivo* (Strick *et al*, 2000) has raised concerns as to the suitability of its widespread use in humans. This property, which genistein shares with apigenin and quercetin, is considered germane to the aetiology of childhood leukaemia, in which dietary Soya as a source of genistein has been implicated. Quercetin is an example of a flavonoid, the early clinical development of which has been aborted because of poor bioavailability (Ferry *et al*, 1996). Compared to genistein and quercetin, tricin lacks such toxicological features, since it has been shown to be incapable of inducing the *MLL* gene cleavage (Borkhard, Mann, Hudson and Gescher, unpublished). Furthermore, three recent studies commissioned by the US National Cancer Institute have established that tricin lacks mutagenic properties as reflected by its complete inability to induce either chromosomal aberrations in Chinese hamster ovary cells (‘Evaluation of tricin in the Chinese hamster ovary chromosome aberration assay’ SRI International technical report, 2003, unpublished), micronuclei in bone marrow erythrocytes of Swiss-Webster mice (‘Evaluation of tricin in the mouse bone marrow micronucleus assay’, SRI International technical report, 2003, unpublished), or revertant colonies in the *Salmonella Escherichia coli* assay (‘Evaluation of tricin in the *Salmonella—E. coli*/microsome preincubation assay, SRI International technical report, 2004, unpublished). The work described here illustrates the considerable growth-inhibitory potency of tricin in MDA-MB-468 cells, but it does not support the notion that tricin as such is a promising candidate for breast cancer chemoprevention. The translation of the growth-inhibitory property into activity in rodent models of breast carcinogenesis *in vivo* may require pharmaceutical optimisation of the formulation of tricin or suitable prodrug development to increase its bioavailability in the target tissue. The high level of tricin found in the gastrointestinal tract after dietary intake renders exploration of its ability to prevent colorectal carcinogenesis propitious. Such attempts are probably worthwhile in the light of the emerging favourable pharmacological profile of tricin, which may ultimately render this flavone a suitable candidate for clinical evaluation.
